# Some Hermite-Hadamard Type Inequalities for Harmonically *s*-Convex Functions

**DOI:** 10.1155/2014/279158

**Published:** 2014-07-24

**Authors:** Feixiang Chen, Shanhe Wu

**Affiliations:** ^1^School of Mathematics and Statistics, Chongqing Three Gorges University, Wanzhou, Chongqing 404000, China; ^2^Department of Mathematics and Computer Science, Longyan University, Longyan, Fujian 364012, China

## Abstract

We establish some estimates of the right-hand side of Hermite-Hadamard type inequalities for functions whose derivatives absolute values are harmonically *s*-convex. Several Hermite-Hadamard type inequalities for products of two harmonically *s*-convex functions are also considered.

## 1. Introduction

Let *f* : *I*⊆*R* → *R* be a convex function and *a*, *b* ∈ *I* with *a* < *b*; then

(1) $$\begin{array}{c} f\left(\frac{a+b}{2}\right)\leq \frac{1}{b-a}\overset{b}{\underset{a}{\int }}f\left(t\right)dt\leq \frac{f\left(a\right)+f\left(b\right)}{2}. \end{array}$$

Inequality (1) is known as the Hermite-Hadamard inequality.

In [1], Hudzik and Maligranda considered the class of functions which are *s*-convex in the second sense. This class of functions is defined as follows.

A function *f* : [0, *∞*)→[0, *∞*) is said to be *s*-convex in the second sense if the inequality

(2) $$\begin{array}{c} f\left(\alpha x+\left(1-\alpha \right)y\right)\leq \alpha^{s}f\left(x\right)+{\left(1-\alpha \right)}^{s}f\left(y\right) \end{array}$$

holds for all *x*, *y* ∈ [0, *∞*), *α* ∈ [0,1] and for some fixed *s* ∈ (0,1].

It can be easily seen that, for *s* = 1, *s*-convexity reduces to ordinary convexity of functions defined on [0, *∞*).

In [2], Dragomir and Fitzpatrick established a variant of Hermite-Hadamard inequality which holds for the *s*-convex functions in the second sense.

**Theorem 1 (see [2]).**

Suppose that *f* : [0, *∞*)→[0, *∞*) is an *s*-convex function in the second sense, where *s* ∈ (0,1] and let *a*, *b* ∈ [0, *∞*), *a* < *b*. If *f* ∈ *L*[*a*, *b*], then the following inequalities hold:

(3) $$\begin{array}{c} 2^{s-1}f\left(\frac{a+b}{2}\right)\leq \frac{1}{b-a}\overset{b}{\underset{a}{\int }}f\left(t\right)dt\leq \frac{f\left(a\right)+f\left(b\right)}{s+1}. \end{array}$$

Some generalizations, improvements, and extensions of inequalities (1) and (3) can be found in the recent papers [2–18].

In [16], İşcan investigated the Hermite-Hadamard type inequalities for harmonically convex functions.

**Definition 2 (see [16]).**

Let *I*⊆*R*∖{0} be a real interval. A function *f* : *I* → *R* is said to be harmonically convex, if

(4) $$\begin{array}{c} f\left(\frac{xy}{tx+\left(1-t\right)y}\right)\leq tf\left(y\right)+\left(1-t\right)f\left(x\right), \end{array}$$

for all *x*, *y* ∈ *I* and *t* ∈ [0,1]. If the inequality in (4) is reversed, then *f* is said to be harmonically concave.

**Theorem 3 (see [16]).**

Let *f* : *I*⊆*R*∖{0} → *R* be a harmonically convex function and *a*, *b* ∈ *I* with *a* < *b*. If *f* ∈ *L*(*a*, *b*), then one has

(5) $$\begin{array}{c} f\left(\frac{2ab}{a+b}\right)\leq \frac{ab}{b-a}\overset{b}{\underset{a}{\int }}\frac{f\left(x\right)}{x^{2}}dx\leq \frac{f\left(a\right)+f\left(b\right)}{2}. \end{array}$$

**Theorem 4 (see [16]).**

Let *f* : *I*⊆*R*∖{0} → *R* be a differentiable function on *I* ^0^ (*I* ^0^ is the interior of *I*), *a*, *b* ∈ *I* with *a* < *b*, and *f*′ ∈ *L*[*a*, *b*]; then

(6) f(a)+f(b)2−abb−a∫abf(x)x2dx =ab(b−a)2∫011−2t(tb+(1−t)a)2f′(abtb+(1−t)a)dt.

In [19], İşcan investigated the Hermite-Hadamard type inequalities for harmonically *s*-convex functions.

**Definition 5 (see [19]).**

Let *I*⊆*R*∖{0} be a real interval. A function *f* : *I*⊆(0, *∞*) → *R* is said to be harmonically *s*-convex, if

(7) $$\begin{array}{c} f\left(\frac{xy}{tx+\left(1-t\right)y}\right)\leq t^{s}f\left(y\right)+{\left(1-t\right)}^{s}f\left(x\right), \end{array}$$

for all *x*, *y* ∈ *I*, *t* ∈ [0,1] and for some fixed *s* ∈ (0,1]. If the inequality in (7) is reversed, then *f* is said to be harmonically *s*-concave.

**Theorem 6 (see [19]).**

Let *f* : *I*⊆(0, *∞*) → *R* be a harmonically *s*-convex function and *a*, *b* ∈ *I* with *a* < *b*. If *f* ∈ *L*(*a*, *b*), then one has

(8) $$\begin{array}{c} 2^{s-1}f\left(\frac{2ab}{a+b}\right)\leq \frac{ab}{b-a}\overset{b}{\underset{a}{\int }}\frac{f\left(x\right)}{x^{2}}dx\leq \frac{f\left(a\right)+f\left(b\right)}{s+1}. \end{array}$$

In [20], Pachpatte established two new Hermite-Hadamard type inequalities for products of convex functions asserted by Theorem 7.

**Theorem 7 (see [20]).**

Let *f* and *g* be real-valued, nonnegative, and convex functions on [*a*, *b*]. Then

(9) 1b−a∫abf(x)g(x)dx≤13M(a,b)+16N(a,b),2f(a+b2)g(a+b2) ≤1b−a∫abf(x)g(x)dx+16M(a,b)+13N(a,b),

where *M*(*a*, *b*) = *f*(*a*)*g*(*a*) + *f*(*b*)*g*(*b*) and *N*(*a*, *b*) = *f*(*a*)*g*(*b*) + *f*(*b*)*g*(*a*).

For more results concerning the Hermite-Hadamard inequality, we refer the reader to [21–25] and the references cited therein.

In this paper, we establish some estimates of the right-hand side of Hermite-Hadamard type inequalities for functions whose derivatives absolute values are harmonically *s*-convex. Moreover, we provide several Hermite-Hadamard type inequalities for products of two harmonically *s*-convex functions.

## 2. Inequalities for Harmonically *s*-Convex Functions

We recall the following special functions.

The gamma function is as follows:

(10) $$\begin{array}{c} \Gamma \left(x\right)=\overset{+\infty }{\underset{0}{\int }}e^{-x}t^{x-1}dt,\;x>0; \end{array}$$

the beta function is as follows:

(11) $$\begin{array}{c} \beta \left(x,y\right)=\overset{1}{\underset{0}{\int }}{\left(1-t\right)}^{y-1}t^{x-1}dt,\;x>0,\;\;y>0,\\ \beta \left(x,y\right)=\frac{\Gamma \left(x\right)\Gamma \left(y\right)}{\Gamma \left(x+y\right)}; \end{array}$$

the hypergeometric function is as follows:

(12) 2F1(x,y;c;z) =1β(y,c−y)∫01ty−1(1−t)c−y−1(1−zt)−xdt,|z|<1, c>y>0.

Our main results are given in the following theorems.

**Theorem 8 .**

Let *f* : *I*⊆(0, *∞*) → *R* be a differentiable function on *I* ^0^ such that *f*′ ∈ *L*[*a*, *b*], where *a*, *b* ∈ *I* ^0^ with *a* < *b*. If |*f*′|^*q*^ is harmonically *s*-convex on [*a*, *b*] for some fixed *s* ∈ (0,1], *q* ≥ 1, then

(13) |f(a)+f(b)2−abb−a∫abf(x)x2dx| ≤ab(b−a)2C11−1/q(a,b)[C2(s;a,b)|f′(b)|q+ C3(s;a,b)|f′(a)|q]1/q,

where

(14) C1(a,b)=b−2(2F1(2,2;3;1−ab)− 2F1(2,1;2;1−ab)+12 2F1(2,1;3;12(1−ab))),C2(s;a,b) =b−2(2s+2 2F1(2,s+2;s+3;1−ab)−1s+1 2F1(2,s+1;s+2;1−ab)+12s(s+1)(s+2) 2F1×(2,s+1;s+3;12(1−ab))),C3(s;a,b)=b−2(2(s+1)(s+2) 2F1(2,2;s+3;1−ab)−1s+1 2F1(2,1;s+2;1−ab)+12 2F1(2,1;3;12(1−ab))).

**Proof**

Let *A*_*t*_ = *ta* + (1 − *t*)*b*. Using Theorem 4, the power mean inequality, and the harmonically *s*-convexity of |*f*′|^*q*^, we have

(15) |f(a)+f(b)2−abb−a∫abf(x)x2dx| ≤ab(b−a)2∫01|1−2t|At2|f′(abAt)|dt ≤ab(b−a)2(∫01|1−2t|At2dt)1−1/q  ×(∫01|1−2t|At2|f′(abAt)|qdt)1/q ≤ab(b−a)2K11−1/q  ×(∫01|1−2t|At2[(1−t)s|f′(a)|q+ts|f′(b)|q]dt)1/q ≤ab(b−a)2K11−1/q(K2|f′(b)|q+K3|f′(a)|q)1/q,

where

(16) K1=∫01|1−2t|At2dt,K2=∫01|1−2t|At2tsdt,K3=∫01|1−2t|At2(1−t)sdt.

Calculating *K*_1_, *K*_2_, and *K*_3_, we find

(17) K1=∫01|1−2t|At2dt=∫01/21−2tAt2dt+∫1/212t−1At2dt=∫012t−1At2dt+2∫01/21−2tAt2dt=2∫01tAt−2dt−∫01At−2dt +∫01(1−u)b−2(1−u12(1−ab))−2du=b−2(2F1(2,2;3;1−ab)− 2F1(2,1;2;1−ab)+12 2F1(2,1;3;12(1−ab)))=C1(a,b).

Similarly, we get

(18) K2=∫01|1−2t|tsAt2dt=∫012t−1At2tsdt+2∫01/21−2tAt2tsdt=2∫01ts+1At−2dt−∫01tsAt−2dt +12s∫01(1−u)usb−2(1−u12(1−ab))−2du=2b−2s+2  2F1(2,s+2;s+3;1−ab) −b−2s+1  2F1(2,s+1;s+2;1−ab) +b−22s(s+1)(s+2) 2F1(2,s+1;s+3;12(1−ab))=C2(s;a,b),

(19) K3=∫01|1−2t|(1−t)sAt2dt=∫012t−1At2(1−t)sdt+2∫01/21−2tAt2(1−t)sdt≤2∫01t(1−t)sAt−2dt −∫01(1−t)sAt−2dt+2∫01/21−2tAt2dt=b−2(2(s+1)(s+2) 2F1(2,2;s+3;1−ab)−1s+1 2F1(2,1;s+2;1−ab)+12 2F1(2,1;3;12(1−ab)))=C3(s;a,b).

This completes the proof of Theorem 8.

**Theorem 9 .**

Let *f* : *I*⊆(0, *∞*) → *R* be a differentiable function on *I* ^0^ such that *f*′ ∈ *L*[*a*, *b*], where *a*, *b* ∈ *I* ^0^ with *a* < *b*. If |*f*′|^*q*^ is harmonically *s*-convex on [*a*, *b*] for some fixed *s* ∈ (0,1], *q* > 1, then

(20) |f(a)+f(b)2−abb−a∫abf(x)x2dx| ≤a(b−a)2b(1p+1)1/p  ×((2F1(2q,s+1;s+2;1−ab)|f′(b)|q+ 2F1(2q,1;s+2;1−ab)|f′(a)|q)    × (s+1)−1)1/q,

where (1/*p*) + (1/*q*) = 1.

**Proof**

Let *A*_*t*_ = *ta* + (1 − *t*)*b*. Utilizing Theorem 4, the Hölder inequality, and the harmonically *s*-convexity of |*f*′|^*q*^, we have

(21) |f(a)+f(b)2−abb−a∫abf(x)x2dx|≤ab(b−a)2∫01|1−2t|At2|f′(abAt)|dt≤ab(b−a)2(∫01|1−2t|pdt)1/p(∫011At2q|f′(abAt)|qdt)1/q≤ab(b−a)2K41/p ×(∫011At2q[(1−t)s|f′(a)|q+ts|f′(b)|q]dt)1/q=ab(b−a)2K41/p[K5|f′(b)|q+K6|f′(a)|q]1/q,

where

(22)  K4=∫01|1−2t|pdt=1p+1, K5=∫01At−2qtsdt =b−2q∫01ts(1−t(1−ab))−2qdt =1(s+1)b2q  2F1(2q,s+1;s+2;1−ab), K6=∫01At−2q(1−t)sdt =b−2q∫01(1−t)s(1−t(1−ab))−2qdt =1(s+1)b2q  2F1(2q,1;s+2;1−ab).

The proof of Theorem 9 is completed.

## 3. Inequalities for Products of Harmonically *s*-Convex Functions

**Theorem 10 .**

Let *f*, *g* : [*a*, *b*]→[0, *∞*), *a*, *b* ∈ (0, *∞*), *a* < *b*, be functions such that *f*, *g*, *fg* ∈ *L*[*a*, *b*]. If *f* is harmonically *s*_1_-convex and *g* is harmonically *s*_2_-convex on [*a*, *b*] for some fixed *s*_1_, *s*_2_ ∈ (0,1], then

(23) abb−a∫abf(x)g(x)x2dx ≤11+s1+s2M(a,b)+Γ(1+s1)Γ(s2+1)Γ(s1+s2+2)N(a,b),

where *M*(*a*, *b*) = *f*(*a*)*g*(*a*) + *f*(*b*)*g*(*b*) and *N*(*a*, *b*) = *f*(*a*)*g*(*b*) + *f*(*b*)*g*(*a*).

**Proof**

Since *f* is harmonically *s*_1_-convex and *g* is harmonically *s*_2_-convex on [*a*, *b*], then for *t* ∈ [0,1] we get

(24) $$\begin{array}{c} f\left(\frac{ab}{ta+\left(1-t\right)b}\right)\leq t^{s_{1}}f\left(b\right)+{\left(1-t\right)}^{s_{1}}f\left(a\right),\\ g\left(\frac{ab}{ta+\left(1-t\right)b}\right)\leq t^{s_{2}}g\left(b\right)+{\left(1-t\right)}^{s_{2}}g\left(a\right). \end{array}$$

From (24), we get

(25) f(abta+(1−t)b)g(abta+(1−t)b) ≤ts1+s2f(b)g(b)+(1−t)s1+s2f(a)g(a)  +ts1(1−t)s2f(b)g(a)+(1−t)s1ts2f(a)g(b).

Integrating both sides of the above inequality with respect to *t* over [0,1], we obtain

(26) ∫01f(abta+(1−t)b)g(abta+(1−t)b)dt =abb−a∫abf(x)g(x)x2dx ≤1s1+s2+1[f(a)g(a)+f(b)g(b)]  +f(a)g(b)∫01(1−t)s1ts2dt  +f(b)g(a)∫01ts1(1−t)s2dt =11+s1+s2M(a,b)+β(1+s1,s2+1)N(a,b).

The proof of Theorem 10 is completed.

**Remark 11 .**

Taking *s*_1_ = *s*_2_ = 1 in Theorem 10, we obtain

(27) $$\begin{array}{c} \frac{ab}{b-a}\overset{b}{\underset{a}{\int }}\frac{f\left(x\right)g\left(x\right)}{x^{2}}dx\leq \frac{1}{3}M\left(a,b\right)+\frac{1}{6}N\left(a,b\right). \end{array}$$

**Remark 12 .**

Choosing *s*_1_ = *s*_2_ = 1 and *g* ≡ 1 in Theorem 10 gives

(28) $$\begin{array}{c} \frac{ab}{b-a}\overset{b}{\underset{a}{\int }}\frac{f\left(x\right)}{x^{2}}dx\leq \frac{f\left(a\right)+f\left(b\right)}{2}, \end{array}$$

which is the right-hand side inequality of (5).

**Theorem 13 .**

Let *f*, *g* : [*a*, *b*]→[0, *∞*), *a*, *b* ∈ (0, *∞*), *a* < *b*, be functions such that *f*, *g*, *fg* ∈ *L*[*a*, *b*]. If *f* is harmonically *s*_1_-convex and *g* is harmonically *s*_2_-convex on [*a*, *b*] for some fixed *s*_1_, *s*_2_ ∈ (0,1], then

(29) 2s1+s2−1f(2aba+b)g(2aba+b) ≤abb−a∫abf(x)g(x)x2dx+M(a,b)Γ(1+s1)Γ(s2+1)Γ(s1+s2+2)  +1s2+s1+1N(a,b),

where *M*(*a*, *b*) = *f*(*a*)*g*(*a*) + *f*(*b*)*g*(*b*) and *N*(*a*, *b*) = *f*(*a*)*g*(*b*) + *f*(*a*)*g*(*b*).

**Proof**

Using the harmonically *s*-convexity of *f* and *g*, we have for all *x*, *y* ∈ [*a*, *b*]

(30) $$\begin{array}{c} f\left(\frac{2xy}{x+y}\right)\leq \frac{f\left(y\right)+f\left(x\right)}{2^{s_{1}}},\\ g\left(\frac{2xy}{x+y}\right)\leq \frac{g\left(y\right)+g\left(x\right)}{2^{s_{2}}}. \end{array}$$

Choosing *x* = *ab*/(*tb* + (1 − *t*)*a*) and *y* = *ab*/(*tb* + (1 − *t*)*a*), we have

(31) f(2aba+b)g(2aba+b) ≤f(ab/(tb+(1−t)a))+f(ab/(ta+(1−t)b))2s1  ×g(ab/(tb+(1−t)a))+g(ab/(ta+(1−t)b))2s2 =12s1+s2[f(abtb+(1−t)a)g(abtb+(1−t)a)+f(abtb+(1−t)a)g(abta+(1−t)b)+f(abta+(1−t)b)g(abtb+(1−t)a)+f(abta+(1−t)b)g(abta+(1−t)b)] ≤12s1+s2[f(abtb+(1−t)a)g(abtb+(1−t)a)+f(abta+(1−t)b)g(abta+(1−t)b)]  +12s1+s2{[ts1f(a)+(1−t)s1f(b)]×[(1−t)s2g(a)+ts2g(b)]+[(1−t)s1f(a)+ts1f(b)]×[ts2g(a)+(1−t)s2g(b)]} =12s1+s2[f(abtb+(1−t)a)g(abtb+(1−t)a)+f(abta+(1−t)b)g(abta+(1−t)b)]  +12s1+s2{[ts1(1−t)s2+(1−t)s1ts2]M(a,b)+[(1−t)s2+s1+ts2+s1]N(a,b)}.

Integrating the resulting inequality with respect to *t* over [0,1], we get

(32) f(2aba+b)g(2aba+b) ≤12s1+s2[∫01f(abtb+(1−t)a)g(abtb+(1−t)a)dt+∫01f(abta+(1−t)b)g(abta+(1−t)b)dt]  +12s1+s2{M(a,b)∫01[ts1(1−t)s2+(1−t)s1ts2]dt+N(a,b)∫01[(1−t)s2+s1+ts2+s1]dt}.

That is,

(33) f(2aba+b)g(2aba+b) ≤12s1+s2−1abb−a∫abf(x)g(x)x2dx  +12s1+s2{M(a,b)∫01[ts1(1−t)s2+(1−t)s1ts2]dt+N(a,b)∫01[(1−t)s2+s1+ts2+s1]dt}.

From

(34) $$\begin{array}{c} \overset{1}{\underset{0}{\int }}\left[t^{s_{1}}{\left(1-t\right)}^{s_{2}}+{\left(1-t\right)}^{s_{1}}t^{s_{2}}\right]dt=2\beta \left(s_{1}+1,s_{2}+1\right),\\ \overset{1}{\underset{0}{\int }}\left[{\left(1-t\right)}^{s_{2}+s_{1}}+t^{s_{2}+s_{1}}\right]dt=\frac{2}{s_{2}+s_{1}+1}, \end{array}$$

we get

(35) 2s1+s2−1f(2aba+b)g(2aba+b) ≤abb−a∫abf(x)g(x)x2dx+M(a,b)β(s1+1,s2+1)  +N(a,b)1s2+s1+1.

This completes the proof of Theorem 13.

**Remark 14 .**

Putting *s*_1_ = *s*_2_ = 1 in Theorem 13 gives

(36) 2f(2aba+b)g(2aba+b) ≤abb−a∫abf(x)g(x)x2dx+16M(a,b)+13N(a,b).

**Remark 15 .**

If we take *s*_1_ = *s*_2_ = 1 and *g* ≡ 1 in Theorem 13, then we obtain

(37) $$\begin{array}{c} 2f\left(\frac{2ab}{a+b}\right)\leq \frac{ab}{b-a}\overset{b}{\underset{a}{\int }}\frac{f\left(x\right)}{x^{2}}dx+\frac{f\left(a\right)+f\left(b\right)}{2}. \end{array}$$
